# Altered Expression of Ribosome Biogenesis Regulators (TP53, C-MYC, FBL, and NCL) in Precursor B-cell Acute Lymphoblastic Leukemia and Neuroblastoma

**DOI:** 10.3390/cimb48010074

**Published:** 2026-01-12

**Authors:** Michalina Horochowska, Dawid Przystupski, Marta Kamińska, Iwona Bil-Lula, Bernarda Kazanowska, Marek Ussowicz

**Affiliations:** 1 Department of Pediatric Bone Marrow Transplantation, Oncology and Hematology, Wroclaw Medical University, 50-556 Wrocław, Poland; m.horochowska@gmail.com (M.H.); dawid.przystupski@umw.edu.pl (D.P.); bernarda.kazanowska@umw.edu.pl (B.K.); 2 Department of Medical Laboratory Diagnostics, Division of Clinical Chemistry and Laboratory Haematology, Wroclaw Medical University, 50-556 Wrocław, Poland; mkaminskakontakt@gmail.com (M.K.); iwona.bil-lula@umw.edu.pl (I.B.-L.)

**Keywords:** ribosome biogenesis, acute lymphoblastic leukemia, neuroblastoma, TP53, C-MYC, NCL, FBL

## Abstract

Background/Objectives: Rapid cellular proliferation, a hallmark of malignancy, requires sustained and elevated protein synthesis, which in turn requires efficient ribosome biogenesis. The aim of this study was to evaluate the expression levels of TP53, C-MYC, FBL, and NCL in pre-B ALL and neuroblastoma tissues compared to healthy bone marrow samples—factors that may carry prognostic significance in pediatric malignancies. Materials and methods: The cohort included 45 pre-B ALL patients, 19 neuroblastoma patients, and 12 healthy bone marrow donors as controls. Total RNA was extracted from bone marrow or tumor samples and cDNA synthesis was performed with the Bio-Rad iScript kit. Quantitative PCR was conducted using SYBR Green chemistry, with GAPDH as the reference gene. Primers targeted TP53, C-MYC, FBL, and NCL, and gene expression was calculated using the 2^−ΔCt^ method. Results: The expression of *C-MYC* and *FBL* was found to be significantly decreased in patients with pre-B ALL in comparison to the healthy control group. *NCL* expression was highest in healthy donors, intermediate in pre-B ALL, and lowest in neuroblastoma. In addition to intergroup comparisons, correlations between gene expression levels were assessed within each diagnostic group. In the pre-B ALL group, a positive correlation was observed between *TP53* and *C-MYC* expression, as well as between *TP53* and both *FBL* and *NCL*. Furthermore, a significant positive correlation was found between *FBL* and *NCL*. In the neuroblastoma group, a statistically significant positive correlation was identified between *C-MYC* and *FBL* expression. In the control group, *TP53* expression was positively correlated with *NCL*, and *FBL* expression showed a significant positive correlation with *NCL*. Conclusions: This study suggests the altered expression of ribosome biogenesis-related genes in pediatric pre-B acute lymphoblastic leukemia and neuroblastoma. The reported dysregulation suggests a disease-associated disruption in nucleolar function and translational regulation and may contribute to oncogenesis through altered ribosomal assembly, protein synthesis, or proliferative signaling.

## 1. Introduction

Rapid cellular proliferation, a hallmark of malignancy, requires sustained and elevated protein synthesis, which in turn requires efficient ribosome biogenesis. Extensive research conducted in the field of oncology over the last 20–30 years suggests that alterations in ribosome biogenesis are crucial for malignant transformation and progression. This process is largely attributed to the dysregulation of oncogenes and tumor suppressor genes, which play critical roles in controlling cell proliferation and survival [1,2].

Ribosome biogenesis is a multistep process localized primarily within the nucleolus and involves the synthesis, processing, and assembly of ribosomal RNA (rRNA) and ribosomal proteins (RPs). One of the fundamental functions of the nucleolus is controlling RNA metabolism and ribosome biogenesis, including rRNA and pre-rRNA synthesis, rRNA processing, assembly of ribosomal units, and maturation. It is a compartment where a group of tandem repeats of rRNA genes are organized into nucleolar organizing regions (NORs) [3]. Human cells contain about 400 copies of the ribosomal RNA genes, which are transcribed in nucleoli by RNA polymerase I (Pol I) to 47S RNA precursor. 47S RNA precursor is modified by pseudouridylation, 2′O-ribosome methylation, and base methylation to become the mature 18S, 5.8S, and 28S rRNAs. 2′O-ribosome methylation at each site is guided by small nucleolar RNAs (snoRNAs). Simultaneously, 5S rRNA is transcribed by RNA polymerase III (Pol III) and RPs are transcribed by polymerase II (Pol II). Bringing up together, rRNAs and RPs construct the large and small subunit of a mature ribosome. Each large 60S subunit consists of 28 S, 5.8S, and 5S RNAs with 47 ribosomal proteins. Each small 40S subunit consists of 18S RNA and 32 ribosomal proteins. Both units are transported from the nucleolus to the cytoplasm where they combine to form 80S ribosome particle. This process requires at least 150 non-ribosomal proteins and at least 70 sno-RNAs [1,4,5,6]. 

Key regulatory molecules of this process, including p53, C-MYC, fibrillarin (FBL), and nucleolin (NCL), have been widely studied in adult malignancies but remain underexplored in pediatric cancers. Importantly, ribosome biogenesis is developmentally regulated, and pediatric tumors arise within a distinct hematopoietic and neurodevelopmental context characterized by higher baseline proliferative flux, different epigenetic states, and age-specific oncogenic programs. These features may modify nucleolar stress signaling and the balance between MYC-driven transcription and p53-mediated checkpoint control compared with adult tumors. Although direct comparative pediatric–adult datasets are limited, emerging developmental biology and pediatric cancer studies suggest that age-dependent regulation of ribosome production can shape tumor phenotypes and therapy response [1,2,6]. Although p53 and C-MYC regulate a broad range of cellular targets, we included them in our analysis because both play pivotal roles in the regulation of ribosome biogenesis. FBL and NCL were selected as representative core effectors of rRNA processing and ribosome assembly, enabling us to interrogate complementary nodes of the biogenesis pathway in a retrospective cohort with limited material. A preliminary study, along with the literature evidence, highlighted the need to investigate these factors in childhood hematological and solid tumors [7]. 

p53 is a master tumor suppressor that functions as a central integrator of cellular stress responses [8,9]. p53 also modulates ribosome biogenesis by interacting with RNA polymerases I and III, thereby repressing rRNA, tRNA, and snoRNA synthesis. In addition to this direct transcriptional repression, ribosome biogenesis is coupled to TP53 activation through the nucleolar stress checkpoint: impaired rRNA production or ribosomal subunit assembly leads to the accumulation of free ribosomal proteins (RP, e.g., RPL5, RPL11, RPL23), which bind and inhibit MDM2, thereby stabilizing p53 and triggering cell cycle arrest or apoptosis [10]. This RP–MDM2–TP53 axis provides a key link between nucleolar function and tumor suppression. Its activity is tightly regulated by the induction of MDM2 expression, an E3 ubiquitin ligase, which in a feedback loop increases p53 degradation in response to ribosomal stress [10].

C-MYC, a well-established proto-oncogene, promotes cellular proliferation by enhancing ribosome production. It activates all three RNA polymerases: boosting rRNA precursor synthesis via RNA polymerase I, increasing 5S RNA transcription via RNA polymerase III, and upregulating ribosomal protein and assembly factor expression via RNA polymerase II. C-MYC therefore drives coordinated increases in rRNA, 5S rRNA, snoRNA-dependent processing, and RP gene transcription. Conversely, several RPs re-leased during nucleolar stress (notably RPL11 and RPL5) can suppress C-MYC activity, forming a negative feedback loop that restrains ribosome output when assembly is perturbed. This bidirectional MYC–RP relationship further integrates growth signaling with the nucleolar checkpoint [11,12].

FBL, a core component of the small nucleolar ribonucleoproteins (snoRNPs), is essential for rRNA methylation and processing. It catalyzes 2′-O-methylation of rRNA, a critical step in ribosome maturation, and influences rRNA transcription via chromatin modification [13,14]. Overexpression of FBL has been associated with enhanced tumor cell proliferation and resistance to therapy [15,16].

NCL is a multifunctional protein involved in rRNA transcription, pre-rRNA processing, and ribosome assembly. Predominantly localized in the dense fibrillar compartment of the nucleolus, NCL interacts with rDNA chromatin and RNA polymerase I to enhance rRNA synthesis [17]. It also participates in cellular signaling and mRNA stabilization, and its dysregulation has been implicated in tumor growth and progression [18,19].

**Figure 1:**
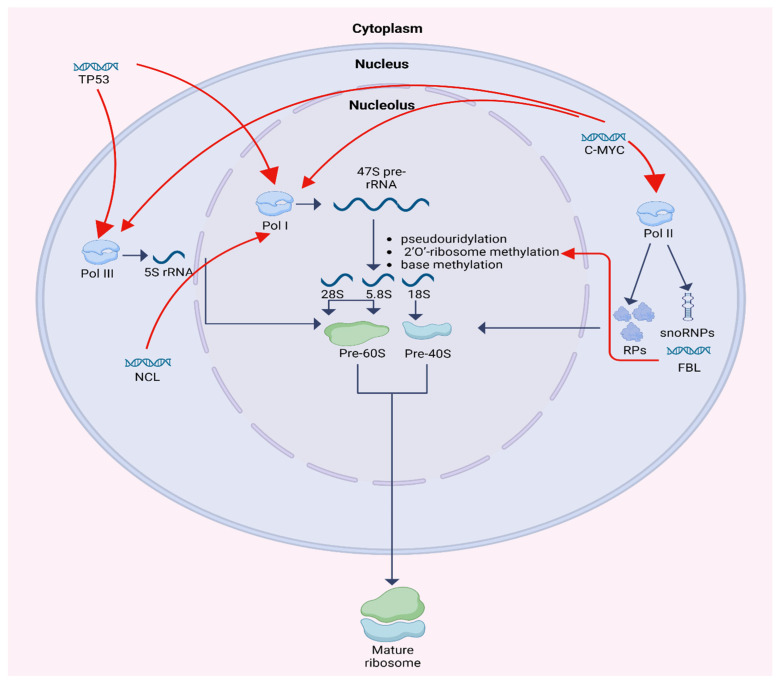
Schematic diagram of ribosome biogenesis and its regulation by TP53, C-MYC, FBL, and NCL. *Created in BioRender. Horochowska, M. (2026) https://BioRender.com/8f0jeli* (accessed in 10 January 2026).

The regulation of ribosome biogenesis is shown in Figure 1.

The expression patterns and interactions of these key ribosome biogenesis factors may carry prognostic significance in pediatric malignancies. However, their roles in childhood pre-B acute lymphoblastic leukemia (pre-B ALL) and neuroblastoma remain largely uncharacterized. The aim of this study was to evaluate the expression levels of TP53, C-MYC, FBL, and NCL in pre-B ALL and neuroblastoma tissues compared to healthy bone marrow samples. We focused on pre-B acute lymphoblastic leukemia and neuroblastoma, which represent the most frequent pediatric hematologic and solid tumors, respectively. Despite their distinct origins, both are marked by rapid cellular proliferation and therefore may depend on enhanced ribosome biogenesis. We also sought to assess correlations between gene expressions, clinical features, and outcomes in order to identify potential biomarkers for prognosis in pediatric oncology.

## 2. Materials and Methods

This research was carried out as a single-center retrospective study within the time frame 2017–2020 at the Department of Peadiatric Bone Marrow Transplantation, Oncology and Hematology, Wroclaw Medical University, Poland, and at the Department of Medical Laboratory Diagnostics, Division of Clinical Chemistry and Laboratory Haematology, Wroclaw Medical University, Poland. The protocol of the study was approved by the Ethics Committee of the Medical University of Wroclaw. Informed consent obtained from patients’ parents was mandatory.

All patients were treated and monitored at our institution. The study cohort consisted of three groups: 45 newly diagnosed patients with precursor B-cell acute lymphoblastic leukemia (pre-B ALL), 19 patients with newly diagnosed neuroblastoma, and 12 healthy bone marrow donors (control group). Bone marrow samples from pre-B ALL patients were collected at the time of diagnosis. Tumor tissue samples from neuroblastoma patients were obtained during initial diagnostic procedures. Control samples were collected from healthy donors prior to bone marrow donation for transplant purposes. Detailed patient characteristics for the pre-B ALL, neuroblastoma and healthy donors groups are presented in Table 1, Table 2 and Table 3, respectively. Cytomolecular characterization was derived from routine diagnostic testing available in the clinical records at diagnosis, including conventional karyotyping and/or targeted assays (e.g., FISH/RT-PCR) for recurrent B ALL alterations and copy number changes (as summarized in Table 1); for neuroblastoma, MYCN amplification was assessed by routine testing when performed (Table 2). Comprehensive DNA-based mutational profiling (including TP53 sequencing) and systematic assessment of 17p/TP53 locus status were not performed in this study because it was a retrospective analysis based on archival RNA with no paired DNA and insufficient residual material to enable uniform genomic testing across all cases.

RNA extraction and reverse transcription–quantitative PCR (RT-qPCR). Mononucleated cells from 57 cryopreserved bone marrow samples were washed in phosphate-buffered saline (PBS) and isolated by Ficoll gradient centrifugation. For neuroblastoma patients, fresh tumor biopsy samples obtained at diagnosis were mechanically dissociated, and total RNA was extracted directly from tumor tissue using the RNeasy Mini Kit (Qiagen, Hilden, Germany) according to the manufacturer’s protocol. Total RNA was extracted using Macherey-Nagel NucleoSpin RNA according to the manufacturer’s instructions. The concentration and purity of the RNA were evaluated with a microvolume ultraviolet (UV) spectrophotometer (NanoDrop Lite, Thermo Scientific, Wilmigton, DE, USA).

Reverse transcription of RNA into cDNA was performed using BioRad iScript cDNA Synthesis Kit cat. 170-8891 according to the manufacturer’s instructions. Quantitative PCR was conducted using a BioRad CFX96 Touch Reat-Time PCR Detection System with GAPDH serving as the reference housekeeping gene. Primer sequences utilized for GADPH were as follows: forward, 5′-GAAGGTGAAGGTCGGAGTC-3′ and reverse, 5′-GAAGATGGTGATGGGATTTC-3′. Primer sequences utilized for TP53 were as follows: forward, 5′-GCGTGTTTGTGCCTGTCCTG-3′ and reverse, 5′-GTGCTCGCTTAGTGCTCCCT-3′ [8], for FBL as follows: forward, 5′-TGGACCAGATCCACATCAAA-3′ and reverse: 5′-GACTAGACCATCCGGACCAA-3′ [20], for NCL as follows: forward, 5′-GCACTTGGAGTGGTGAATCAAA-3′ and reverse, 5′-AAATGATACCCTTTAGATTTGCC-3′ [21], and for C-MYC as follows: forward: 5′-GCTGCTTAGACGCTGGATTTTT-3′ and reverse: 5′-ACCGAGTCGTAGTCGAGGTCAT-3′ [22]. The reaction mix (20 µL final volume) consisted of iTag Universal Sybr Green Supermix with ROX (BioRad) 10 µL, forward and reverse primers (500 nM final conc.), cDNA (100 ng), and water up to 20 µL. All reactions were performed in triplicate. Relative gene expression was calculated using the 2^−ΔCt^ method with GAPDH as the internal control.

Statistical analysis and data presentation were performed using Statistica 13 software (TIBCO Software Inc. 2017, STATISTICA, version 13, Dell, OK, USA). The following statistical methods were used: Kruskal–Wallis test with Dunn’s post hoc test (ANOVA) for comparison of gene expressions between disease groups, Spearman’s rank correlation coefficient for evaluating correlations between gene expression levels and continuous variables, and Mann–Whitney U test with continuity correction for logistic regression for correlations between gene expressions and imaging findings. All results were considered significant if the *p*-value was less than 0.05.

## 3. Results

When comparing gene expression across the three groups, no statistically significant differences in TP53 expression were observed. However, the expression of C-MYC was found to be significantly decreased in patients with pre-B ALL in comparison to the healthy control group (*p* < 0.001). No significant differences in C-MYC expression were noted between the neuroblastoma and control groups, nor between the pre-B ALL and neuroblastoma groups. A similar pattern was observed for FBL expression, which was significantly lower in the pre-B ALL group compared to the controls (*p* = 0.004), while no significant differences were seen between neuroblastoma and the other groups. For NCL, expression was highest in healthy donors, intermediate in pre-B ALL, and lowest in neuroblastoma. The differences in NCL expression were statistically significant between neuroblastoma and pre-B ALL (*p* = 0.003), between neuroblastoma and the controls (*p* < 0.001), and borderline significant between pre-B ALL and the controls (*p* = 0.051). Based on the group median 2^−ΔCt^ values, NCL expression in pre-B ALL vs. controls was ~1.48-fold lower (≈ 1.5-fold), in neuroblastoma vs. pre-B ALL, it was 2.21-fold lower (≈ 2.2-fold), and in neuroblastoma vs. controls, it was 3.27-fold lower (≈ 3.3-fold).

**Figure 2:**
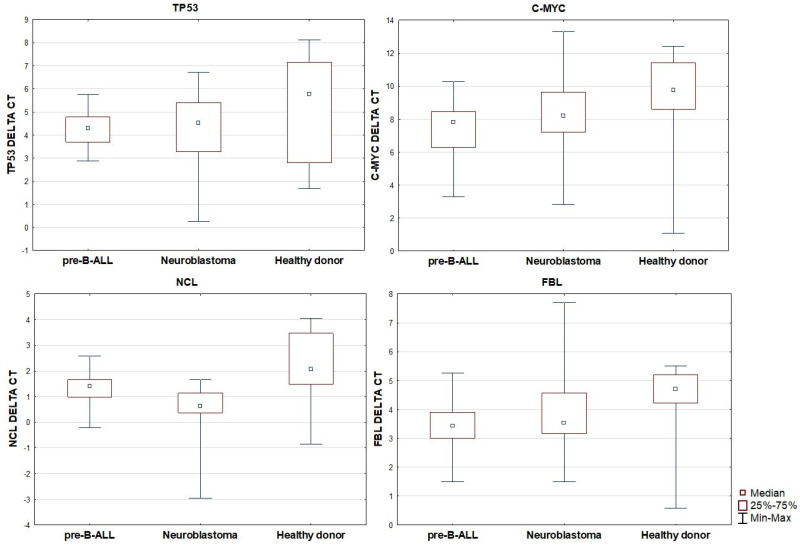
Relative expression of TP53, C-MYC, NCL, and FBL in pre-B ALL, neuroblastoma, and healthy patients.

These results are summarized in Table 4 and shown in Figure 2.

Comparison of FBL gene expression showed similar results.

In addition to intergroup comparisons, correlations between gene expression levels were assessed within each diagnostic group using Spearman’s rank correlation coefficient (Table 5). In the pre-B ALL group, a positive correlation was observed between TP53 and C-MYC expression (*p* = 0.042), as well as between TP53 and both FBL and NCL (*p* < 0.001 for both). Furthermore, a significant positive correlation was found between FBL and NCL (*p* < 0.001). Other pairwise comparisons within this group did not reach statistical significance.

In the neuroblastoma group, a statistically significant positive correlation was identified between C-MYC and FBL expression (*p* = 0.004), while no other correlations were found to be significant (Table 6). 

In the control group, TP53 expression was positively correlated with NCL (*p* < 0.001), and FBL expression showed a significant positive correlation with NCL (*p* = 0.033) (Table 7).

Further analyses were conducted to explore potential associations between gene expression levels and clinical or laboratory parameters. No statistically significant correlations were found between gene expression levels and clinical, laboratory, or treatment-related parameters in the pre-B ALL group. This included blood counts, biochemical markers, organ involvement, CNS status, treatment response, risk stratification, and relapse. Similarly, in the neuroblastoma group, TP53, C-MYC, FBL, and NCL expression showed no significant associations with disease stage, histopathological subtype, or MYCN amplification. Associations between TP53, C-MYC, FBL, and NCL expression and B ALL cytomolecular features (Table 1) were not formally tested because several subgroups were small and incompletely characterized, limiting statistical power and increasing the risk of spurious findings.

## 4. Discussion

Our study demonstrated diverse expression patterns of ribosomal biogenesis genes between malignant and non-malignant tissues, and uncovered several gene–gene expression correlations that may reflect underlying biological mechanisms. The most striking finding was the significant downregulation of C-MYC and FBL in pre-B ALL compared to healthy controls. Although C-MYC overexpression is well-documented in many malignancies, our finding of reduced C-MYC expression in pre-B ALL contrasts with previous reports demonstrating elevated C-MYC levels in B-cell ALL. However, it is important to highlight that most of those studies focused on adult patients or on cases with high-risk ALL subtypes, such as Philadelphia chromosome-positive (Ph+) ALL or those harboring IKZF1 (Ikaros) mutations, which may account for the observed discrepancies [23,24,25]. It is important to note that many studies reporting C-MYC overexpression in B-ALL have utilized the NALM-6 cell line, which was derived from a young adult with B-ALL. As such, this model may not be fully representative of pediatric disease biology and could potentially bias the interpretation of gene expression patterns in childhood B-ALL [23,26].

A positive correlation between TP53 and C-MYC expression was observed in pre-B ALL patients, while in neuroblastoma, C-MYC expression was positively associated with FBL. Although our data suggest a positive relationship between TP53 and C-MYC in pre-B ALL, previous studies have primarily described TP53 as a negative regulator of C-MYC. In both mouse and human cell models, TP53 has been shown to downregulate C-MYC expression as part of its role in inducing G1 cell cycle arrest. This downregulation is believed to occur through direct binding of p53 to the C-MYC promoter, although there is no consensus on the precise localization of the binding site. Additionally, TP53 has been shown to repress C-MYC transcription via histone deacetylation, further reducing transcriptional activity at the C-MYC locus [27,28]. A positive correlation between TP53 and C-MYC expression in the pre-B ALL group contrasts with studies in adult malignancies where p53 commonly suppresses C-MYC via transcriptional repression or microRNA intermediates such as miR-145 [29,30]. 

Because our study didn’t show any significant differences in TP53 in pre-B ALL or in neuroblastoma in comparison to the control group, we concluded that C-MYC’s expression may be controlled in a TP53-independent manner. One of those mechanisms is an inhibition of C-MYC by L11 ribosomal protein through a negative feedback-inhibitory mechanism. The activity of C-MYC is downregulated by overexpression of L11 ribosomal protein and upregulated after reduction of L11 by small interfering RNA. What’s more, C-MYC itself is able to induce L11 ribosomal protein, thereby forming a negative autoregulatory feedback loop [31,32]. 

FBL regulation by p53 through direct binding or indirectly as response to small interfering RNAs has been previously established [33,34]. The suppression of FBL by p53 led to modifications in rRNA methylation pattern at a single nucleotide level, which has been shown to impair translational fidelity and reprogram protein synthesis in favor of IRES-dependent translation of oncogenic transcripts such as C-MYC may reflect altered upstream signaling or leukemia-specific transcriptional landscapes [14,35].

The positive correlation between C-MYC and FBL expression in neuroblastoma further supports the established role of C-MYC in enhancing ribosome biogenesis through upregulation of ribosomal RNA modification enzymes and assembly factors [20,36]. It is conceivable that in neuroblastoma, MYC-driven transcriptional programs remain more intact or active, particularly in MYCN-negative cases, where C-MYC may act as a compensatory driver [37].

A notable observation was the progressive reduction in NCL expression from controls to pre-B ALL to neuroblastoma, with the lowest levels observed in neuroblastoma patients. In neuroblastoma, reduced NCL levels may reflect advanced tumor loss of differentiation or aberrant nucleolar function. Additionally, the strong correlation between TP53 and NCL in both pre-B ALL and control groups reinforces the bidirectional regulatory relationship proposed in previous studies. It has been discovered that NCL is able to interact with p53 5’UTR, stabilize its stem-loop structure and reduce p53 level in a dose-responsive manner by reduction of translation and on a basis of this discovery may be considered to be an oncogene [38]. What’s more, in healthy cells which don’t undergo stress, NCL promotes base-pairing of the 5’ and 3’UTR of TP53 mRNA. Subsequently NCL occupies the 5’CS/3’CS double-stranded region and stops the translation process. In stress conditions NCL is outcompeted by RPL26 and the translation is able to take place [39,40]. 

On the basis of cellular stress, after an increase of p53 concentration, the NCL-p53 complex is formed and transported to the nucleoplasm where the NCL can interact with replication protein A (RPA), a protein that binds to single-stranded DNA. RPA is essential for DNA metabolism, including its replication, repair, and damage signaling [41,42]. What’s more, NCL is able to bind to MDM2, an E3 ubiquitin ligase of TP53, reduce its level and hereby leads to an increase of p53 in cells which are not under stress conditions [43,44]. In addition, NCL can suppress MDM2 autoubiquitination and stabilize the MDM2 protein [45]. In consequence, NCL is known to bind and stabilize TP53 mRNA under certain stress conditions, while also modulating MDM2 levels, thereby influencing p53 turnover and activity [42,46]. Finally, no significant associations were found between gene expression and clinical or laboratory parameters, treatment response, or prognostic indicators in either pre-B ALL or neuroblastoma. While this may reflect biological independence, it is also possible that the prognostic significance of these markers requires a larger cohort to reach statistical power. Alternatively, the lack of correlation could be due to post-transcriptional regulation or differences in protein expression and functional activity not captured at the mRNA level.

Nonetheless, our study has limitations. It is retrospective and single-center, with a limited sample size, particularly in the neuroblastoma group. First, the pre-B ALL cohort likely comprises multiple cytomolecular subtypes with distinct biology, because subtype data were not uniformly available and subgroup sizes were small, we could not perform robust subtype-stratified analyses. Second, the healthy bone marrow controls had limited clinical annotation and may differ in age from the pediatric cancer cohorts; given that ribosome biogenesis and C-MYC/p53 networks are developmentally regulated, age mismatch could confound intergroup comparisons. Third, blast involvement in ALL samples ranged from 50–99%, and neuroblastoma tumor cell content could not be standardized across cases. Because RNA was extracted from bulk mononuclear marrow or tumor tissue without malignant-cell enrichment, measured expression may partially reflect non-tumor components. This concern is amplified by the use of bone marrow controls for comparison with solid-tumor neuroblastoma, where the non-malignant microenvironment differs qualitatively from marrow. mRNA levels were used as surrogates for functional protein expression, which may not fully reflect biological activity. Mutation status of TP53 and potential variants in the other analyzed genes were not assessed in this cohort, and along with chimeric transcriptional regulation, alternative splicing, and post-translational modifications, remain important limitations of our study. Future studies of this pathway should include protein-level validation (e.g., Western blot or immunohistochemistry). Our analysis was limited to a restricted set of ribosome biogenesis regulators. A more comprehensive approach including additional nucleolar proteins and ribosomal proteins would likely provide further mechanistic insight. It should also be noted that our gene panel did not include several ribosome biogenesis–related signature genes that have recently been recognized as relevant in pediatric B-ALL, such as RPL9, and RPS15A [46,47]. Due to the retrospective design and limited RNA material, we were unable to extend our analysis to these targets. Nevertheless, their reported importance highlights promising directions for future studies in larger, prospective cohorts.

## 5. Conclusions

Our study suggests altered expression of ribosome biogenesis-related genes in pediatric pre-B acute lymphoblastic leukemia and neuroblastoma, which may warrant further validation in larger, independent cohorts with protein-level and functional analyses. The reported dysregulation is consistent with an association between these malignancies and perturbed nucleolar/ribosome biogenesis programs; however, given the retrospective design, bulk tissue sampling, and RNA-only approach, the data should be interpreted as exploratory and hypothesis-generating rather than evidence of causality. Future prospective studies integrating cytomolecular stratification, age-matched controls, tumor cell enrichment, and proteomic/functional readouts are required to define whether these regulators contribute to disease mechanisms or have clinical utility. Despite these dysregulations, the majority of patients in this study demonstrated clinical response and favorable outcome, suggesting the efficacy of treatment protocols in minimizing the effects of these molecular alterations. In this limited cohort, expression changes were not associated with clinical risk features or outcome, but the study was not powered to detect subtle prognostic effects. In addition, targeting ribosome biogenesis may offer benefits, particularly for patients with treatment resistance or relapse. At present, this therapeutic perspective remains speculative in our setting and should be addressed in dedicated functional and translational studies. In conclusion, while ribosome biogenesis dysregulation may reflect underlying tumor biology, it does not necessarily preclude successful treatment outcomes. Future studies integrating genomic, proteomic, and functional data will be able to fully define the clinical utility of ribosome biogenesis markers as prognostic tools or therapeutic targets.

## Figures and Tables

**Table 1:** Characteristics of acute lymphoblastic leukemia patients.

Patients’ Characteristics—Pre-B ALL	
Sex	23 male (51%), 22 female (49%)
Age of diagnosis	1–18 yrs, median 5 yrs
White blood count	0.8–228.0 K/uL, median 9.86 K/uL
Hemoglobin	3.0–12.1 g/dL, median 8.3 g/dL
Platelets	5–429 K/ul, median 54 K/ul
Lactate dehydrogenase	144.0–3325.0 U/L, median 530 U/L
Hepatosplenomegaly	Yes—34 pts (76%) No—11 pts (24%)
Lymphadenopathy	Yes—20 pts (44%) No—25 pts (56%)
Involvement of the mediastinum	Yes—6 pts (13%) No—39 pts (87%)
Involvement of testicles	Yes—1 pts (4%) No—22 (96%)
Blast count in the bone marrow	50–99%, median 94%
CNS status	CNS 1—28 pts (62%) CNS 2—14 pts (31%) CNS 3—3 pts (7%)
Prednisone response	PGR—42 (93%) PPR—3 (7%)
Bone marrow blast count on day 15	M1—41 pts (91%) M2—3 pts (7%) M3—1 pts (2%)
FCM-MRD% on day 15	0.01–64.4%, median 0.25%
Bone marrow blast count on day 33	M1—45 pts (100%)
Cytomolecular subtype—ploidy	Normal—32 pts (72%) Hiperdiploidy—6 pts (13%) Hipertriploidy—2 pts (4%) Hipodiploidy—1 pts (2%) Hipotriploidy—1 pts (2%) Data not available—3 pts (7%)
Cytomolecular subtype—ETV6/RUNX1 fusion gene	7 pts (16%)
Cytomolecular subtype—CDKN2A heterozygous deletion	3 pts (7%)
Cytomolecular subtype—other genetic mutation	9 pts (20%)
Risk group	SRG—17 pts (38%) Early non-HRG—1 pts (2%) MRG—24 pts (53%) HRG—3 pts (7%)
Relapse	Relapse—3 pts (7%) No relapse—42 pts (93%)

Legend: yrs—years old, pts—patients, blasts—immature blood cells in bone marrow, CNS status—the presence of blasts in initial CSF before start of chemotherapy, prednisone response—evaluated on day 8 after 7 days of prednisone treatment in peripheral blood, PGR—prednisone good response (absolute blast count in peripheral blood < 1000/μL), PPR—prednisone poor response (absolute blast count in peripheral blood ≥ 1000/μL), day 15—investigation of cytomorphological response in bone marrow on protocol day 15, M1—<5% blast cells in bone marrow, M2—≥5% and <25% blasts in bone marrow, M3—≥25% blasts in bone marrow, FCM-MRD%—investigation of minimal residual disease by flow cytometry on protocol day 15, day 33—investigation of cytomorphological response in bone marrow on protocol day 33, SRG—standard risk group, early non-HRG—early non-high-risk group, MRG—medium-risk group, HRG—high-risk group.

**Table 2:** Characteristics of neuroblastoma patients.

Patients’ Characteristics—Neuroblastoma	
Sex	12 male pts (63%) 7 female pts (37%)
Histopathological diagnosis	NBL—8 pts (42%) iNBL—4 pts (21%) GNBL—3 pts (16%) GN—4 pts (21%)
INSS stage	1—5 pts (26%) 3—3 pts (16%) 4—11 pts (58%)
MYCN amplification	positive—5 pts (26.5%) negative—9 pts (47%) no data—5 pts (25.5%)

Legend: pts—patients, yrs—years old, NBL—neuroblastoma, iNBL—infant neuroblastoma, GN—ganglioneuroma, GNBL—ganglioneuroblastoma, INSS—The International Neuroblastoma Staging System (INSS).

**Table 3:** Characteristics of healthy donor patients.

Patients’ Characteristics—Healthy Donors	
Sex	8 male pts (67%) 4 female pts (33%)
Age	5–53 yrs, median 8.5 yrs (9 pts < 18 yrs, 3 pts > 18yrs)

Legend: pts—patients, yrs—years old.

**Table 4:** The correlation between gene expression levels across pre-B ALL, neuroblastoma, and healthy control groups.

		Delta Median	Pre-B ALL	Neuroblastoma	Healthy Donor
TP53	pre-B ALL	4.3	-	1.000	0.328
	Neuroblastoma	4.53	1.000	-	0.637
	Healthy Donor	5.78	0.328	0.637	-
C-MYC	pre-B ALL	7.82	-	0.302	<0.001 *
	Neuroblastoma	8.21	0.302	-	0.204
	Healthy Donor	9.75	<0.001 *	0.204	-
FBL	pre-B ALL	4.43	-	0.580	0.004 *
	Neuroblastoma	3.54	0.580	-	0.198
	Healthy Donor	4.70	0.004 *	0.198	-
NCL	pre-B ALL	1.39	-	0.003 *	0.051
	Neuroblastoma	0.63	0.003 *	-	<0.001 *
	Healthy Donor	2.06	0.051	<0.001 *	-

Statistically significant results are shown in bold and marked with an asterisk.

**Table 5:** Results of Spearman’s rank correlation coefficient between expressions of the analyzed genes in pre-B ALL patients.

Gene	TP53	C-MYC	FBL	NCL
TP53	-	*p* = 0.042 *	*p* < 0.001 *	*p* < 0.001 *
C-MYC	0.30 *	-	*p* = 0.258	*p* = 0.452
FBL	0.72 *	0.17	-	*p* < 0.001 *
NCL	0.71 *	-	0.73 *	-

The lower part of the table shows Spearman’s rho and the upper part the *p*-values. Statistically significant results are shown in bold and marked with an asterisk.

**Table 6:** Results of Spearman’s rank correlation coefficient between expressions of the analyzed genes in neuroblastoma patients.

Gene	TP53	C-MYC	FBL	NCL
TP53	-	*p* = 0.390	*p* = 0.508	*p* = 0.050
C-MYC	0.21	-	*p* = 0.004 *	*p* = 0.094
FBL	0.16	0.63 *	-	*p* = 0.299
NCL	0.45	0.39	0.25	-

The lower part of the table shows Spearman’s rho and the upper part the *p*-values. Statistically significant results are shown in bold and marked with an asterisk.

**Table 7:** Results of Spearman’s rank correlation coefficient between expressions of the analyzed genes in healthy patients (control group).

Gene	TP53	C-MYC	FBL	NCL
TP53	-	*p* = 0.497	*p* = 0.112	*p* < 0.001 *
C-MYC	−0.21	-	*p* = 0.664	*p* = 0.871
FBL	0.48	0.14	-	*p* = 0.033 *
NCL	0.88 *	−0.05	0.61 *	-

The lower part of the table shows Spearman’s rho and the upper part the *p*-values. Statistically significant results are shown in bold and marked with an asterisk.

## Data Availability

The original contributions presented in this study are included in the article. Further inquiries can be directed to the corresponding author.
